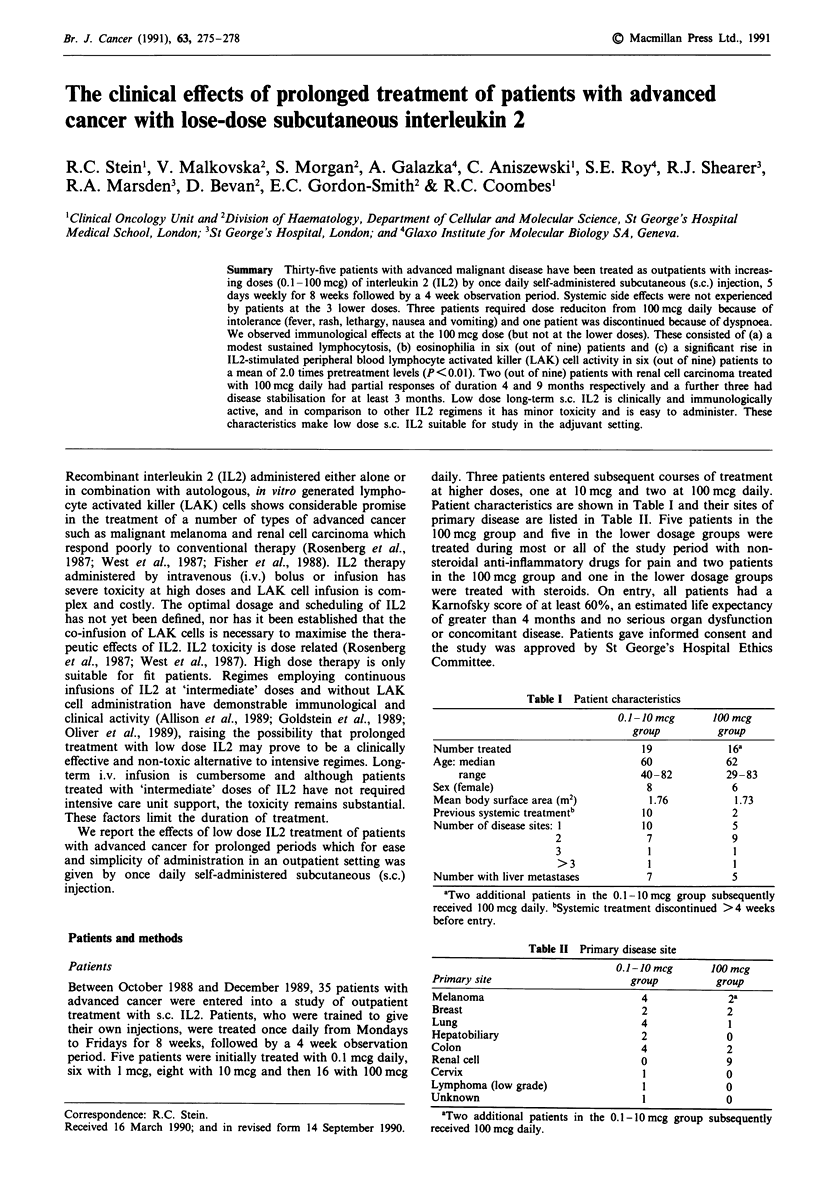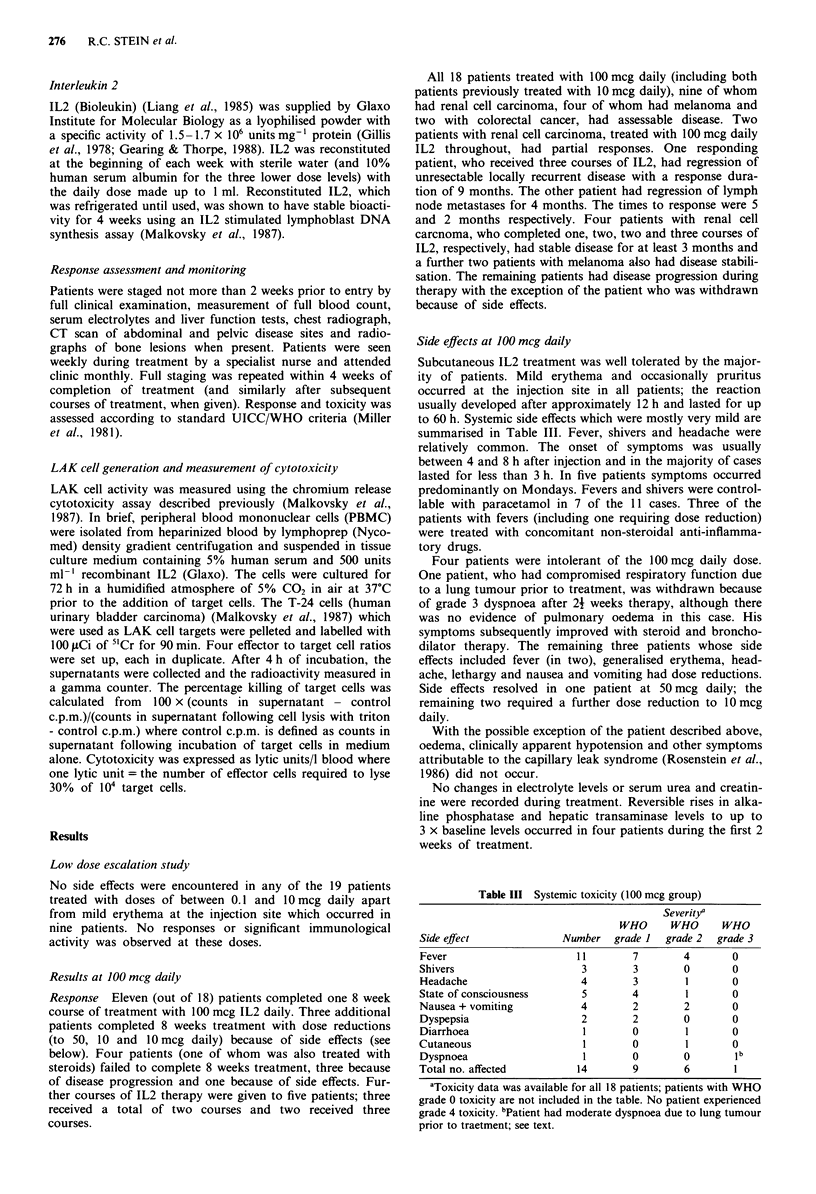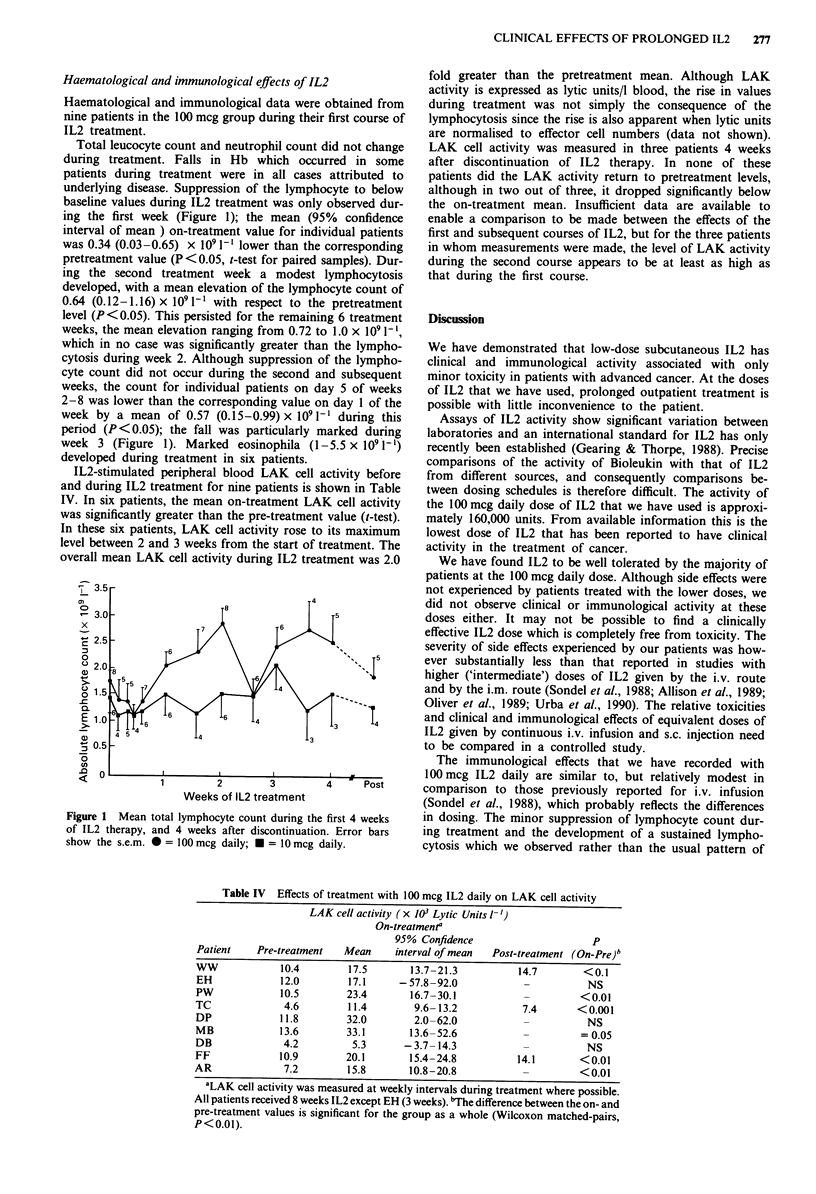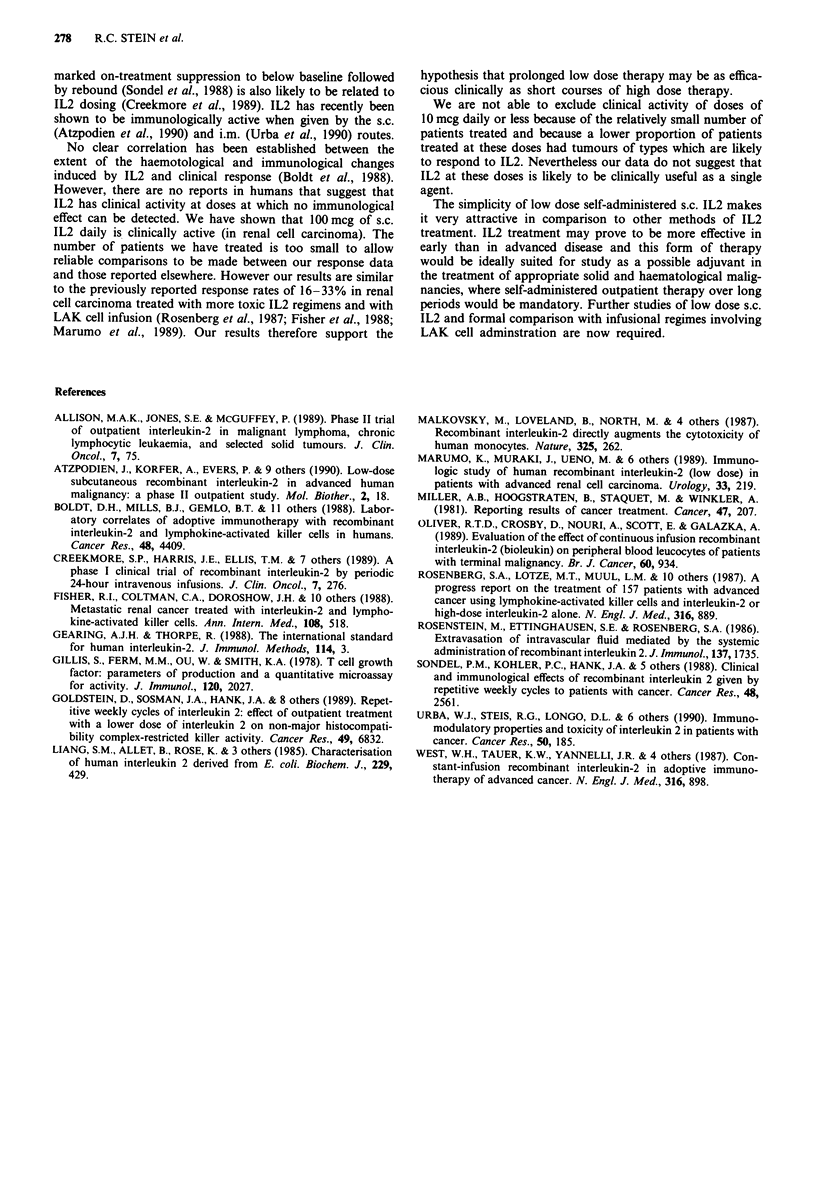# The clinical effects of prolonged treatment of patients with advanced cancer with low-dose subcutaneous interleukin-2 [corrected]

**DOI:** 10.1038/bjc.1991.64

**Published:** 1991-02

**Authors:** R. C. Stein, V. Malkovska, S. Morgan, A. Galazka, C. Aniszewski, S. E. Roy, R. J. Shearer, R. A. Marsden, D. Bevan, E. C. Gordon-Smith

**Affiliations:** Clinical Oncology Unit, St. George's Hospital, Medical School, London.

## Abstract

Thirty-five patients with advanced malignant disease have been treated as outpatients with increasing doses (0.1-100 mcg) of interleukin 2 (IL2) by once daily self-administered subcutaneous (s.c.) injection, 5 days weekly for 8 weeks followed by a 4 week observation period. Systemic side effects were not experienced by patients at the 3 lower doses. Three patients required dose reduction from 100 mcg daily because of intolerance (fever, rash, lethargy, nausea and vomiting) and one patient was discontinued because of dyspnoea. We observed immunological effects at the 100 mcg dose (but not at the lower doses). These consisted of (a) a modest sustained lymphocytosis, (b) eosinophilia in six (out of nine) patients and (c) a significant rise in IL2-stimulated peripheral blood lymphocyte activated killer (LAK) cell activity in six (out of nine) patients to a mean of 2.0 times pretreatment levels (P less than 0.01). Two (out of nine) patients with renal cell carcinoma treated with 100 mcg daily had partial responses of duration 4 and 9 months respectively and a further three had disease stabilisation for at least 3 months. Low dose long-term s.c. IL2 is clinically and immunologically active, and in comparison to other IL2 regimens it has minor toxicity and is easy to administer. These characteristics make low dose s.c. IL2 suitable for study in the adjuvant setting.


 © Macmillan Press Ltd., 1991

### The clinical effects of prolonged treatment of patients with advanced cancer with lose-dose subcutaneous interleukin 2

R.C. Stein[1], V. Malkovska[2], S. Morgan[2], A. Galazka[4], C. Aniszewski[1], S.E. Roy[4], R.J. Shearer[3], R.A. Marsden[3], D. Bevan[2], E.C. Gordon-Smith[2] & R.C. Coombes[1]

[1]*Clinical Oncology Unit and* [2]*Division of Haematology, Department of Cellular and Molecular Science, St George's Hospital Medical School, London;* [3]*St George's Hospital, London; and* [4]*Glaxo Institute for Molecular Biology SA, Geneva.*

**Summary** Thirty-five patients with advanced malignant disease have been treated as outpatients with increasing doses (0.1-100 mcg) of interleukin 2 (IL2) by once daily self-administered subcutaneous (s.c.) injection, 5 days weekly for 8 weeks followed by a 4 week observation period. Systemic side effects were not experienced by patients at the 3 lower doses. Three patients required dose reduciton from 100 mcg daily because of intolerance (fever, rash, lethargy, nausea and vomiting) and one patient was discontinued because of dyspnoea. We observed immunological effects at the 100 mcg dose (but not at the lower doses). These consisted of (a) a modest sustained lymphocytosis, (b) eosinophilia in six (out of nine) patients and (c) a significant rise in IL2-stimulated peripheral blood lymphocyte activated killer (LAK) cell activity in six (out of nine) patients to a mean of 2.0 times pretreatment levels ($P < 0.01$). Two (out of nine) patients with renal cell carcinoma treated with 100 mcg daily had partial responses of duration 4 and 9 months respectively and a further three had disease stabilisation for at least 3 months. Low dose long-term s.c. IL2 is clinically and immunologically active, and in comparison to other IL2 regimens it has minor toxicity and is easy to administer. These characteristics make low dose s.c. IL2 suitable for study in the adjuvant setting.

Recombinant interleukin 2 (IL2) administered either alone or in combination with autologous, *in vitro* generated lymphocyte activated killer (LAK) cells shows considerable promise in the treatment of a number of types of advanced cancer such as malignant melanoma and renal cell carcinoma which respond poorly to conventional therapy (Rosenberg *et al.*, 1987; West *et al.*, 1987; Fisher *et al.*, 1988). IL2 therapy administered by intravenous (i.v.) bolus or infusion has severe toxicity at high doses and LAK cell infusion is complex and costly. The optimal dosage and scheduling of IL2 has not yet been defined, nor has it been established that the co-infusion of LAK cells is necessary to maximise the therapeutic effects of IL2. IL2 toxicity is dose related (Rosenberg *et al.*, 1987; West *et al.*, 1987). High dose therapy is only suitable for fit patients. Regimes employing continuous infusions of IL2 at 'intermediate' doses and without LAK cell administration have demonstrable immunological and clinical activity (Allison *et al.*, 1989; Goldstein *et al.*, 1989; Oliver *et al.*, 1989), raising the possibility that prolonged treatment with low dose IL2 may prove to be a clinically effective and non-toxic alternative to intensive regimes. Long-term i.v. infusion is cumbersome and although patients treated with 'intermediate' doses of IL2 have not required intensive care unit support, the toxicity remains substantial. These factors limit the duration of treatment.

We report the effects of low dose IL2 treatment of patients with advanced cancer for prolonged periods which for ease and simplicity of administration in an outpatient setting was given by once daily self-administered subcutaneous (s.c.) injection.

### Patients and methods

### *Patients*

Between October 1988 and December 1989, 35 patients with advanced cancer were entered into a study of outpatient treatment with s.c. IL2. Patients, who were trained to give their own injections, were treated once daily from Mondays to Fridays for 8 weeks, followed by a 4 week observation period. Five patients were initially treated with 0.1 mcg daily, six with 1 mcg, eight with 10 mcg and then 16 with 100 mcg daily. Three patients entered subsequent courses of treatment at higher doses, one at 10 mcg and two at 100 mcg daily. Patient characteristics are shown in Table I and their sites of primary disease are listed in Table II. Five patients in the 100 mcg group and five in the lower dosage groups were treated during most or all of the study period with non-steroidal anti-inflammatory drugs for pain and two patients in the 100 mcg group and one in the lower dosage groups were treated with steroids. On entry, all patients had a Karnofsky score of at least 60%, an estimated life expectancy of greater than 4 months and no serious organ dysfunction or concomitant disease. Patients gave informed consent and the study was approved by St George's Hospital Ethics Committee.

**Table I** Patient characteristics

| | *0.1-10 mcg group* | *100 mcg group* |
|---|---|---|
| Number treated | 19 | 16[a] |
| Age: median | 60 | 62 |
| range | 40-82 | 29-83 |
| Sex (female) | 8 | 6 |
| Mean body surface area ($m^2$) | 1.76 | 1.73 |
| Previous systemic treatment[b] | 10 | 2 |
| Number of disease sites: 1 | 10 | 5 |
| 2 | 7 | 9 |
| 3 | 1 | 1 |
| >3 | 1 | 1 |
| Number with liver metastases | 7 | 5 |

[a]Two additional patients in the 0.1-10 mcg group subsequently received 100 mcg daily. [b]Systemic treatment discontinued >4 weeks before entry.

**Table II** Primary disease site

| *Primary site* | *0.1-10 mcg group* | *100 mcg group* |
|---|---|---|
| Melanoma | 4 | 2[a] |
| Breast | 2 | 2 |
| Lung | 4 | 1 |
| Hepatobiliary | 2 | 0 |
| Colon | 4 | 2 |
| Renal cell | 0 | 9 |
| Cervix | 1 | 0 |
| Lymphoma (low grade) | 1 | 0 |
| Unknown | 1 | 0 |

[a]Two additional patients in the 0.1-10 mcg group subsequently received 100 mcg daily.

Correspondence: R.C. Stein.
Received 16 March 1990; and in revised form 14 September 1990.

*Interleukin 2*

IL2 (Bioleukin) (Liang *et al.*, 1985) was supplied by Glaxo Institute for Molecular Biology as a lyophilised powder with a specific activity of 1.5–1.7 × $10^6$ units $mg^{-1}$ protein (Gillis *et al.*, 1978; Gearing & Thorpe, 1988). IL2 was reconstituted at the beginning of each week with sterile water (and 10% human serum albumin for the three lower dose levels) with the daily dose made up to 1 ml. Reconstituted IL2, which was refrigerated until used, was shown to have stable bioactivity for 4 weeks using an IL2 stimulated lymphoblast DNA synthesis assay (Malkovsky *et al.*, 1987).

*Response assessment and monitoring*

Patients were staged not more than 2 weeks prior to entry by full clinical examination, measurement of full blood count, serum electrolytes and liver function tests, chest radiograph, CT scan of abdominal and pelvic disease sites and radiographs of bone lesions when present. Patients were seen weekly during treatment by a specialist nurse and attended clinic monthly. Full staging was repeated within 4 weeks of completion of treatment (and similarly after subsequent courses of treatment, when given). Response and toxicity was assessed according to standard UICC/WHO criteria (Miller *et al.*, 1981).

*LAK cell generation and measurement of cytotoxicity*

LAK cell activity was measured using the chromium release cytotoxicity assay described previously (Malkovsky *et al.*, 1987). In brief, peripheral blood mononuclear cells (PBMC) were isolated from heparinized blood by lymphoprep (Nycomed) density gradient centrifugation and suspended in tissue culture medium containing 5% human serum and 500 units $ml^{-1}$ recombinant IL2 (Glaxo). The cells were cultured for 72 h in a humidified atmosphere of 5% $CO_2$ in air at 37°C prior to the addition of target cells. The T-24 cells (human urinary bladder carcinoma) (Malkovsky *et al.*, 1987) which were used as LAK cell targets were pelleted and labelled with 100 μCi of $^{51}Cr$ for 90 min. Four effector to target cell ratios were set up, each in duplicate. After 4 h of incubation, the supernatants were collected and the radioactivity measured in a gamma counter. The percentage killing of target cells was calculated from 100 × (counts in supernatant – control c.p.m.)/(counts in supernatant following cell lysis with triton - control c.p.m.) where control c.p.m. is defined as counts in supernatant following incubation of target cells in medium alone. Cytotoxicity was expressed as lytic units/l blood where one lytic unit = the number of effector cells required to lyse 30% of $10^4$ target cells.

### Results

*Low dose escalation study*

No side effects were encountered in any of the 19 patients treated with doses of between 0.1 and 10 mcg daily apart from mild erythema at the injection site which occurred in nine patients. No responses or significant immunological activity was observed at these doses.

*Results at 100 mcg daily*

*Response* Eleven (out of 18) patients completed one 8 week course of treatment with 100 mcg IL2 daily. Three additional patients completed 8 weeks treatment with dose reductions (to 50, 10 and 10 mcg daily) because of side effects (see below). Four patients (one of whom was also treated with steroids) failed to complete 8 weeks treatment, three because of disease progression and one because of side effects. Further courses of IL2 therapy were given to five patients; three received a total of two courses and two received three courses.

All 18 patients treated with 100 mcg daily (including both patients previously treated with 10 mcg daily), nine of whom had renal cell carcinoma, four of whom had melanoma and two with colorectal cancer, had assessable disease. Two patients with renal cell carcinoma, treated with 100 mcg daily IL2 throughout, had partial responses. One responding patient, who received three courses of IL2, had regression of unresectable locally recurrent disease with a response duration of 9 months. The other patient had regression of lymph node metastases for 4 months. The times to response were 5 and 2 months respectively. Four patients with renal cell carcnoma, who completed one, two, two and three courses of IL2, respectively, had stable disease for at least 3 months and a further two patients with melanoma also had disease stabilisation. The remaining patients had disease progression during therapy with the exception of the patient who was withdrawn because of side effects.

*Side effects at 100 mcg daily*

Subcutaneous IL2 treatment was well tolerated by the majority of patients. Mild erythema and occasionally pruritus occurred at the injection site in all patients; the reaction usually developed after approximately 12 h and lasted for up to 60 h. Systemic side effects which were mostly very mild are summarised in Table III. Fever, shivers and headache were relatively common. The onset of symptoms was usually between 4 and 8 h after injection and in the majority of cases lasted for less than 3 h. In five patients symptoms occurred predominantly on Mondays. Fevers and shivers were controllable with paracetamol in 7 of the 11 cases. Three of the patients with fevers (including one requiring dose reduction) were treated with concomitant non-steroidal anti-inflammatory drugs.

Four patients were intolerant of the 100 mcg daily dose. One patient, who had compromised respiratory function due to a lung tumour prior to treatment, was withdrawn because of grade 3 dyspnoea after 2½ weeks therapy, although there was no evidence of pulmonary oedema in this case. His symptoms subsequently improved with steroid and bronchodilator therapy. The remaining three patients whose side effects included fever (in two), generalised erythema, headache, lethargy and nausea and vomiting had dose reductions. Side effects resolved in one patient at 50 mcg daily; the remaining two required a further dose reduction to 10 mcg daily.

With the possible exception of the patient described above, oedema, clinically apparent hypotension and other symptoms attributable to the capillary leak syndrome (Rosenstein *et al.*, 1986) did not occur.

No changes in electrolyte levels or serum urea and creatinine were recorded during treatment. Reversible rises in alkaline phosphatase and hepatic transaminase levels to up to 3 × baseline levels occurred in four patients during the first 2 weeks of treatment.

**Table III** Systemic toxicity (100 mcg group)

| *Side effect* | *Number* | Severity[a] *WHO grade 1* | *WHO grade 2* | *WHO grade 3* |
|---|---|---|---|---|
| Fever | 11 | 7 | 4 | 0 |
| Shivers | 3 | 3 | 0 | 0 |
| Headache | 4 | 3 | 1 | 0 |
| State of consciousness | 5 | 4 | 1 | 0 |
| Nausea + vomiting | 4 | 2 | 2 | 0 |
| Dyspepsia | 2 | 2 | 0 | 0 |
| Diarrhoea | 1 | 0 | 1 | 0 |
| Cutaneous | 1 | 0 | 1 | 0 |
| Dyspnoea | 1 | 0 | 0 | 1[b] |
| Total no. affected | 14 | 9 | 6 | 1 |

[a]Toxicity data was available for all 18 patients; patients with WHO grade 0 toxicity are not included in the table. No patient experienced grade 4 toxicity. [b]Patient had moderate dyspnoea due to lung tumour prior to traetment; see text.

*Haematological and immunological effects of IL2*

Haematological and immunological data were obtained from nine patients in the 100 mcg group during their first course of IL2 treatment.

Total leucocyte count and neutrophil count did not change during treatment. Falls in Hb which occurred in some patients during treatment were in all cases attributed to underlying disease. Suppression of the lymphocyte to below baseline values during IL2 treatment was only observed during the first week (Figure 1); the mean (95% confidence interval of mean ) on-treatment value for individual patients was 0.34 (0.03–0.65) $\times 10^9 l^{-1}$ lower than the corresponding pretreatment value ($P < 0.05$, *t*-test for paired samples). During the second treatment week a modest lymphocytosis developed, with a mean elevation of the lymphocyte count of 0.64 (0.12–1.16) $\times 10^9 l^{-1}$ with respect to the pretreatment level ($P < 0.05$). This persisted for the remaining 6 treatment weeks, the mean elevation ranging from 0.72 to 1.0 $\times 10^9 l^{-1}$, which in no case was significantly greater than the lymphocytosis during week 2. Although suppression of the lymphocyte count did not occur during the second and subsequent weeks, the count for individual patients on day 5 of weeks 2–8 was lower than the corresponding value on day 1 of the week by a mean of 0.57 (0.15–0.99) $\times 10^9 l^{-1}$ during this period ($P < 0.05$); the fall was particularly marked during week 3 (Figure 1). Marked eosinophila (1–5.5 $\times 10^9 l^{-1}$) developed during treatment in six patients.

IL2-stimulated peripheral blood LAK cell activity before and during IL2 treatment for nine patients is shown in Table IV. In six patients, the mean on-treatment LAK cell activity was significantly greater than the pre-treatment value (*t*-test). In these six patients, LAK cell activity rose to its maximum level between 2 and 3 weeks from the start of treatment. The overall mean LAK cell activity during IL2 treatment was 2.0

**Figure 1** Mean total lymphocyte count during the first 4 weeks of IL2 therapy, and 4 weeks after discontinuation. Error bars show the s.e.m. ● = 100 mcg daily; ■ = 10 mcg daily.

fold greater than the pretreatment mean. Although LAK activity is expressed as lytic units/l blood, the rise in values during treatment was not simply the consequence of the lymphocytosis since the rise is also apparent when lytic units are normalised to effector cell numbers (data not shown). LAK cell activity was measured in three patients 4 weeks after discontinuation of IL2 therapy. In none of these patients did the LAK activity return to pretreatment levels, although in two out of three, it dropped significantly below the on-treatment mean. Insufficient data are available to enable a comparison to be made between the effects of the first and subsequent courses of IL2, but for the three patients in whom measurements were made, the level of LAK activity during the second course appears to be at least as high as that during the first course.

### Discussion

We have demonstrated that low-dose subcutaneous IL2 has clinical and immunological activity associated with only minor toxicity in patients with advanced cancer. At the doses of IL2 that we have used, prolonged outpatient treatment is possible with little inconvenience to the patient.

Assays of IL2 activity show significant variation between laboratories and an international standard for IL2 has only recently been established (Gearing & Thorpe, 1988). Precise comparisons of the activity of Bioleukin with that of IL2 from different sources, and consequently comparisons between dosing schedules is therefore difficult. The activity of the 100 mcg daily dose of IL2 that we have used is approximately 160,000 units. From available information this is the lowest dose of IL2 that has been reported to have clinical activity in the treatment of cancer.

We have found IL2 to be well tolerated by the majority of patients at the 100 mcg daily dose. Although side effects were not experienced by patients treated with the lower doses, we did not observe clinical or immunological activity at these doses either. It may not be possible to find a clinically effective IL2 dose which is completely free from toxicity. The severity of side effects experienced by our patients was however substantially less than that reported in studies with higher ('intermediate') doses of IL2 given by the i.v. route and by the i.m. route (Sondel *et al.*, 1988; Allison *et al.*, 1989; Oliver *et al.*, 1989; Urba *et al.*, 1990). The relative toxicities and clinical and immunological effects of equivalent doses of IL2 given by continuous i.v. infusion and s.c. injection need to be compared in a controlled study.

The immunological effects that we have recorded with 100 mcg IL2 daily are similar to, but relatively modest in comparison to those previously reported for i.v. infusion (Sondel *et al.*, 1988), which probably reflects the differences in dosing. The minor suppression of lymphocyte count during treatment and the development of a sustained lymphocytosis which we observed rather than the usual pattern of

**Table IV** Effects of treatment with 100 mcg IL2 daily on LAK cell activity

| | LAK cell activity ($\times 10^3$ Lytic Units $l^{-1}$) | | | | |
|---|---|---|---|---|---|
| | | On-treatment[a] | | | |
| *Patient* | *Pre-treatment* | *Mean* | *95% Confidence interval of mean* | *Post-treatment* | *P (On-Pre)*[b] |
| WW | 10.4 | 17.5 | 13.7–21.3 | 14.7 | <0.1 |
| EH | 12.0 | 17.1 | –57.8–92.0 | – | NS |
| PW | 10.5 | 23.4 | 16.7–30.1 | – | <0.01 |
| TC | 4.6 | 11.4 | 9.6–13.2 | 7.4 | <0.001 |
| DP | 11.8 | 32.0 | 2.0–62.0 | – | NS |
| MB | 13.6 | 33.1 | 13.6–52.6 | – | = 0.05 |
| DB | 4.2 | 5.3 | –3.7–14.3 | – | NS |
| FF | 10.9 | 20.1 | 15.4–24.8 | 14.1 | <0.01 |
| AR | 7.2 | 15.8 | 10.8–20.8 | – | <0.01 |

[a]LAK cell activity was measured at weekly intervals during treatment where possible. All patients received 8 weeks IL2 except EH (3 weeks). [b]The difference between the on- and pre-treatment values is significant for the group as a whole (Wilcoxon matched-pairs, $P < 0.01$).

marked on-treatment suppression to below baseline followed by rebound (Sondel *et al.*, 1988) is also likely to be related to IL2 dosing (Creekmore *et al.*, 1989). IL2 has recently been shown to be immunologically active when given by the s.c. (Atzpodien *et al.*, 1990) and i.m. (Urba *et al.*, 1990) routes.

No clear correlation has been established between the extent of the haemotological and immunological changes induced by IL2 and clinical response (Boldt *et al.*, 1988). However, there are no reports in humans that suggest that IL2 has clinical activity at doses at which no immunological effect can be detected. We have shown that 100 mcg of s.c. IL2 daily is clinically active (in renal cell carcinoma). The number of patients we have treated is too small to allow reliable comparisons to be made between our response data and those reported elsewhere. However our results are similar to the previously reported response rates of 16–33% in renal cell carcinoma treated with more toxic IL2 regimens and with LAK cell infusion (Rosenberg *et al.*, 1987; Fisher *et al.*, 1988; Marumo *et al.*, 1989). Our results therefore support the hypothesis that prolonged low dose therapy may be as efficacious clinically as short courses of high dose therapy.

We are not able to exclude clinical activity of doses of 10 mcg daily or less because of the relatively small number of patients treated and because a lower proportion of patients treated at these doses had tumours of types which are likely to respond to IL2. Nevertheless our data do not suggest that IL2 at these doses is likely to be clinically useful as a single agent.

The simplicity of low dose self-administered s.c. IL2 makes it very attractive in comparison to other methods of IL2 treatment. IL2 treatment may prove to be more effective in early than in advanced disease and this form of therapy would be ideally suited for study as a possible adjuvant in the treatment of appropriate solid and haematological malignancies, where self-administered outpatient therapy over long periods would be mandatory. Further studies of low dose s.c. IL2 and formal comparison with infusional regimes involving LAK cell adminstration are now required.

### References

ALLISON, M.A.K., JONES, S.E. & MCGUFFEY, P. (1989). Phase II trial of outpatient interleukin-2 in malignant lymphoma, chronic lymphocytic leukaemia, and selected solid tumours. *J. Clin. Oncol.*, **7**, 75.

ATZPODIEN, J., KORFER, A., EVERS, P. & 9 others (1990). Low-dose subcutaneous recombinant interleukin-2 in advanced human malignancy: a phase II outpatient study. *Mol. Biother.*, **2**, 18.

BOLDT, D.H., MILLS, B.J., GEMLO, B.T. & 11 others (1988). Laboratory correlates of adoptive immunotherapy with recombinant interleukin-2 and lymphokine-activated killer cells in humans. *Cancer Res.*, **48**, 4409.

CREEKMORE, S.P., HARRIS, J.E., ELLIS, T.M. & 7 others (1989). A phase I clinical trial of recombinant interleukin-2 by periodic 24-hour intravenous infusions. *J. Clin. Oncol.*, **7**, 276.

FISHER, R.I., COLTMAN, C.A., DOROSHOW, J.H. & 10 others (1988). Metastatic renal cancer treated with interleukin-2 and lymphokine-activated killer cells. *Ann. Intern. Med.*, **108**, 518.

GEARING, A.J.H. & THORPE, R. (1988). The international standard for human interleukin-2. *J. Immunol. Methods*, **114**, 3.

GILLIS, S., FERM, M.M., OU, W. & SMITH, K.A. (1978). T cell growth factor: parameters of production and a quantitative microassay for activity. *J. Immunol.*, **120**, 2027.

GOLDSTEIN, D., SOSMAN, J.A., HANK, J.A. & 8 others (1989). Repetitive weekly cycles of interleukin 2: effect of outpatient treatment with a lower dose of interleukin 2 on non-major histocompatibility complex-restricted killer activity. *Cancer Res.*, **49**, 6832.

LIANG, S.M., ALLET, B., ROSE, K. & 3 others (1985). Characterisation of human interleukin 2 derived from *E. coli. Biochem. J.*, **229**, 429.

MALKOVSKY, M., LOVELAND, B., NORTH, M. & 4 others (1987). Recombinant interleukin-2 directly augments the cytotoxicity of human monocytes. *Nature*, **325**, 262.

MARUMO, K., MURAKI, J., UENO, M. & 6 others (1989). Immunologic study of human recombinant interleukin-2 (low dose) in patients with advanced renal cell carcinoma. *Urology*, **33**, 219.

MILLER, A.B., HOOGSTRATEN, B., STAQUET, M. & WINKLER, A. (1981). Reporting results of cancer treatment. *Cancer*, **47**, 207.

OLIVER, R.T.D., CROSBY, D., NOURI, A., SCOTT, E. & GALAZKA, A. (1989). Evaluation of the effect of continuous infusion recombinant interleukin-2 (bioleukin) on peripheral blood leucocytes of patients with terminal malignancy. *Br. J. Cancer*, **60**, 934.

ROSENBERG, S.A., LOTZE, M.T., MUUL, L.M. & 10 others (1987). A progress report on the treatment of 157 patients with advanced cancer using lymphokine-activated killer cells and interleukin-2 or high-dose interleukin-2 alone. *N. Engl. J. Med.*, **316**, 889.

ROSENSTEIN, M., ETTINGHAUSEN, S.E. & ROSENBERG, S.A. (1986). Extravasation of intravascular fluid mediated by the systemic administration of recombinant interleukin 2. *J. Immunol.*, **137**, 1735.

SONDEL, P.M., KOHLER, P.C., HANK, J.A. & 5 others (1988). Clinical and immunological effects of recombinant interleukin 2 given by repetitive weekly cycles to patients with cancer. *Cancer Res.*, **48**, 2561.

URBA, W.J., STEIS, R.G., LONGO, D.L. & 6 others (1990). Immunomodulatory properties and toxicity of interleukin 2 in patients with cancer. *Cancer Res.*, **50**, 185.

WEST, W.H., TAUER, K.W., YANNELLI, J.R. & 4 others (1987). Constant-infusion recombinant interleukin-2 in adoptive immunotherapy of advanced cancer. *N. Engl. J. Med.*, **316**, 898.